# O.4.5-2 Participation in organized and unorganized sports in adolescence: associations with physical activity levels in adulthood

**DOI:** 10.1093/eurpub/ckad133.210

**Published:** 2023-09-11

**Authors:** Tuuli Suominen, Katja Pahkala, Suvi Rovio, Xiaolin Young, Janne Kulmala, Mirja Hirvensalo, Olli Raitakari, Tuija Tammelin, Kasper Salin

**Affiliations:** Faculty of Sport and Health Sciences, University of Jyväskylä, Jyväskylä, Finland; Research Centre of Applied and Preventive Cardiovascular Medicine, University of Turku, Turku, Finland; Centre for Population Health Research, University of Turku and Turku University Hospital, Turku, Finland; Paavo Nurmi Centre and Unit for Health and Physical Activity, University of Turku, Turku, Finland; Research Centre of Applied and Preventive Cardiovascular Medicine, University of Turku, Turku, Finland; Centre for Population Health Research, University of Turku and Turku University Hospital, Turku, Finland; Likes, School of Health and Social Studies, Jamk University of Applied Sciences, Jyväskylä, Finland; Likes, School of Health and Social Studies, Jamk University of Applied Sciences, Jyväskylä, Finland; Faculty of Sport and Health Sciences, University of Jyväskylä, Jyväskylä, Finland; Research Centre of Applied and Preventive Cardiovascular Medicine, University of Turku, Turku, Finland; Centre for Population Health Research, University of Turku and Turku University Hospital, Turku, Finland; Department of Clinical Physiology and Nuclear Medicine, Turku University Hospital, University of Turku, Turku, Finland; tuuli.suominen@jyu.fi; Likes, School of Health and Social Studies, Jamk University of Applied Sciences, Jyväskylä, Finland; Faculty of Sport and Health Sciences, University of Jyväskylä, Jyväskylä, Finland

## Abstract

**Purpose:**

Participation in organized youth sports (OYS) has been linked to higher levels of physical activity (PA) in adulthood. However, the longitudinal associations of OYS and PA compared to unorganized sports and non-participation have not been extensively studied. This study aims to explore the associations of both organized and unorganized sports in adolescence with PA levels in midlife.

**Methods:**

Participants in this study were drawn from the on-going, population-based prospective Young Finns Study. Data from follow-ups conducted in the years 1980, 1983, 1986, and 1989 were utilized to divide the participants into groups of active OYS, active unorganized sports, and non-participants, separately at the ages of 9 (n = 548), 12 (n = 727), 15 (n = 752), and 18 (n = 767). The groups were formed according to self-reported frequency and intensity of leisure time PA and participation in sports club activities in adolescence. Accelerometer-derived PA (mean daily minutes of sedentary time [ST], light-intensity PA [LPA], and moderate-to-vigorous PA [MVPA]) in midlife was assessed in 2018-2020. Differences in adult PA between the youth PA groups were analyzed using analysis of covariance, separately in males and females.

**Results:**

Compared to non-participants, boys who actively participated in OYS at age 9 had lower ST (mean difference [SE]: -38 [15], p = 0.045) and higher LPA (29 [12], p = 0.050) in midlife. No significant group differences were observed for girls at age 9 or for either sex at age 12 or 15, or for boys at age 18. In girls at age 18, significant group differences were found in MVPA in midlife in favor of both organized and unorganized sports compared to non-participants (15 [3], p < 0.001; 8 [3], p = 0.014, respectively).

**Conclusions:**

Active participation in organized sports in boys at age 9 and active participation in both organized and unorganized sports in girls at age 18 associated with higher levels of physical activity in midlife. These findings suggest that promoting both types of sports participation in adolescence may contribute to the development of a physically active lifestyle across the life course.

**Support/Funding Source:**

The work was supported by the Ministry of Education and Culture.

